# Extracellular Superoxide Dismutase Activity and Plasma Malondialdehyde in Human Immunodeficiency Virus Subjects of Kano State as Surrogate Markers of CD_4_ Status

**Published:** 2010-12

**Authors:** Muhammad Yalwa Gwarzo, Surajo Al-Kassim Muhammad

**Affiliations:** 1 *Department of Chemical Pathology, Bayero University Kano, PMB 3011, Kano, Nigeria;*; 2 *Department of Chemical Pathology and Immunology, Aminu Kano Teaching Hospital PMB 3452 Kano, Nigeria*

**Keywords:** oxidative Stress, CD_4_, Malondialdehyde (MDA), Vitamin A, Human Immunodeficiency Virus (HIV), (Extracellular) superoxide dismutase

## Abstract

This study looked at the profile of plasma extracellular superoxide dismutase (SOD3) activity, malondialdehyde (MDA) vis-à-vis that of CD_4_ counts in human immunodeficiency virus subjects in Kano State, Nigeria. The subjects for this study comprised twenty (20) non-HIV infected volunteers as control and one hundred (100) HIV infected subjects. Forty nine (49) infected patients have not been on treatment, while fifty one (51) were at various stages of treatment. There was a negative correlation between the serum malondialdehyde concentration and CD_4_ count (Pearson r=−0.68, *p*<0.01). There was also a negative correlation between serum malondialdehyde concentration and extracellular superoxide dismutase activity ((Pearson r=−0.71, *p*<0.01) and Vitamin A concentration (Pearson r=−0.75; *p*<0.01). Conversely a positive correlation was observed between the CD_4_ counts in HIV infected patients and activity of extracellular superoxide dismutase (Pearson r=0.86, *p*<0.01). Similarly there was a positive correlation between CD_4_ count and serum vitamin A concentration (Pearson r=0.89 *p*<0.01). The possibility remains for using these indicators to monitor HIV patients not eligible for therapy in resource constrained facilities of our rural areas.

## INTRODUCTION

Human Immunodeficiency Virus (HIV) Infection induces a wide array of immunological alterations resulting in a slow but progressive development of opportunistic infections and malignancy, culminating in a disease condition known as acquired immunodeficient syndrome (AIDS) due the collapse of the immune system (1). The mechanism contributing to this progression is thought to be oxidative stress induced by the production of reactive oxygen species (ROS), which plays a critical role in the stimulation of HIV replication and the development of immunodeficiency (2, 3).

Reactive oxygen species activate the nuclear transcription factor NF-Kβ, which is obligatory transcriptional factor for HIV replication (4). This in turns leads to increased viral replication in the CD_4_ T-cells. Thus, the progressive loss of CD_4_ lymphocytes in HIV-infected patients may be related to an oxidative stress induced apoptosis (5).

Estimation of products of ROS such as superoxide anion and peroxidation represents some of the means of determining oxidative stress. Polyunsaturated fatty acids, which are major components of cell membranes, are targets of free radicals attack, producing lipid peroxidation products like malondialdehyde (MDA), and 4-hydroxynonenal (2, 3, 6). Under normal circumstances, the body is protected from such damage by a careful balance between pro-oxidants and antioxidants. The antioxidant system consists of enzymes such as superoxide dismutase, glutathione peroxidase, catalase; and scavenging small molecules endogenously produced such as reduced glutathione (GSH), and those exogenously taken such as vitamin C and vitamin E (7).

Several lines of evidence suggest that HIV infected patients are under chronic oxidative stress. Oxidative stress occurs in HIV infection even at the early stages of disease (8). This pro-oxidant state is a result of an imbalance between the generation of reactive oxygen and the antioxidant system, which is responsible for scavenging these oxidants and ensuring little or no interaction with cellular (8).

Antiretroviral treatment was embarked in Nigeria as a self sponsored program in 1990s, available to only those who could afford. Subsequently government intervention became evident in 2002, with the ambition of providing drug to 10,000 adult and 5,000 children within a year at the cost of $7 per person (9). Large turnout of patients for enrolment in 2004 was a major setback of the program due to lack of commensurate financial commitment for patients’ treatment with antiretroviral drug (9). Out of the 2.7 million HIV patients in Nigeria, 550,000 were eligible for antiretroviral treatment in 2006. Participation by foreign donors such as the U.S. President’s Emergency Plan for AIDS Relief (PEPFAR), the Global Fund and the World Bank (10) has assisted greatly to increase the treatment coverage from 81,000 people (15% of those in need) to 198,000 (26%) by the end of 2007. Since success for the management of HIV disease is not only achieved through provision of drugs, but also constant laboratory monitoring among other things, it is thought possible that simple biochemical analysis can be adopted in the resource constrained health laboratory facilities in monitoring the condition of these patients who could not have access to sophisticated facilities in the tertiary health institution. This has become necessary in view of the fact that the financial brunt of the monitoring is borne entirely by the patients. The gross national income per capita (11) of an average Nigerian is $1940 in 2008. Hence the majority of the patients could not pay for the monitoring test because of the competing demands of other social service for the meager resource. This work looks at the potential of using antioxidant enzymes and product of lipid peroxidation as surrogate markers of immunological failure, in order to provide a cheap and affordable means of providing monitoring service in resource constrained facilities in the secondary health centers.

## MATERIALS AND METHODS

All chemicals were purchased form Sigma Aldrich USA unless otherwise stated.

### Ethical approval and informed consent

This study was conducted according to the guidelines laid down in the Declaration of Helsinki (12) and all procedures involving human subjects/patients were approved by AMINU KANO Teaching Hospital ethical committee, Kano State, Nigeria, where the research was conducted. Similarly, both written and oral informed consent from the subjects recruited for the research was sought before samples were collected.

### Subjects

Forty-Nine HIV infected patients who have not been placed on Anti retroviral therapy and fifty one HIV infected patients who are receiving anti retroviral therapy were recruited for the study. Twenty HIV negative volunteers were included as control.

### Blood Collection

Five milliters (5 cm^3^) of blood sample was collected into EDTA vacutainer tubes from the subject, attending Prof. S. S. Wali centre of Human Virology, Aminu Kano Teaching Hospital, Kano, Nigeria. The whole blood was used to determine CD_4_ count, while the remaining sample was centrifuge to obtain the plasma at 300 rpm for 5 minute. The plasma in sample bottle was wrapped with Aluminum foil and kept at −80°C before analysis.

**Determination of CD_4_ count level:** the CD_4_ count was determined by flow cytometry technique, using Cy-Flow Counter machine (PARTEC, GmbH, Germany).

**Determination of Serum Vitamin A Level:** The method of Carr-Price (13) was used to determine Vitamin A. retinoyl acetate obtained from Sigma (St Lious USA) was used to construct a calibration curve. This was carried out according to the method of Carr and Price (1926). To a stoppered centrifuge tube, 0.5 cm^3^ plasma was added followed by the addition of 0.5 cm^3^ ethanolic potassium hydroxide (containing 1 M potassium hydroxide and 90% absolute ethanol) to precipitate protein. Light petroleum 3.0 cm^3^ of petroleum ether was added and shaken vigorously for 10 min, then centrifuged at a low speed (1000 rpm) for about 1 min. 3.0 cm^3^ of petroleum ether layer was pipitted into a 10 cm^3^ test-tube and evaporated to dryness at 60°C in a water bath with the addition of 0.1 cm^3^ acetic anhydride to remove traces of water. 1 cm^3^ of chloroform was added to the residue, followed by the addition of 3 cm^3^ of antimony trichloride (250 g/L in chloroform). The colour developed rapidly reaching a clearly defined maximum in 5 to 15 seconds and faded quickly. The spectrophotometer was set to zero and reading taken immediately at 620 nm.

Blank was prepared by pipetting 1 cm^3^ of chloroform, 0.1 cm^3^ acetic anhydride, followed by the addition of 3 cm^3^ of antimony trichloride (250 g/L in chloroform).

Vitamin A. Vitamin A as Retinoyl palmitate 1,600,000 usp units per gram purchased from sigma was used to construct a calibration curve. Both working and standard solutions were prepared in absolute ethanol. The standards were treated as test samples. Beckman coulter DU 720 UV/Vis Sepectrophotometer was used to measure absorbance at 620 nm.

### Determination of Plasma Malondialdehyde (MDA)

Plasma malondialdehyde was measured by the method of Ohakawa *et al*. (14). Lipid peroxidation generate peroxide intermediates which upon cleavage release MDA, a product which reacts with Thiobarbitutic Acid (TBA). The product of the reaction is coloured complex, which absorbs light at 532nm

**Methods:** 0.20 cm^3^ plasma was put in a test tube containing 3.0 cm^3^ of glacial acetic acid to which 3.0 cm^3^ of 1% TBA in 2% NaOH was added. The mixture was placed in boiling water for 15min, allowed to cool and the absorbance of the pink coloured product was read at 532 nm. Calibration curve was constructed using malondialdehyde tetrabutylammonium salt obtained from Sigma (St Lious USA).

**Extracellular Superoxide Dismutase (ESOD):** The method of McCord and Fridrich (15) was employed to determine ESOD. The assay protocol consists of two components: superoxide generator and superoxide detector. The generator produces the radical at a controlled rate. Superoxide ion was generated by bubbling oxygen in a solution containing Flavine adenine dinucleotide (FAD) and Ethylene diaminetetraacetatic (EDTA). EDTA donates electron to FAD, which in turns donates to molecular oxygen to produce superoxide radical (generator). Nitroblue tetrazolium salt acts as a scavenger for the free radical (detector).

Superoxide generator

$$\text{EDTA}\;+\;\text{FAD}\;\to \;{\text{EDTA}}^{.}\;+\;{\text{FAD}}^{.}$$

$${\text{FAD}}^{.}+{\text{O}}_{2}\to \text{FAD}+{\text{O}}_{2}.$$

Superoxide Acceptor

O_2_^.^ + Nitroblue tetrazolium salt (NB; yellow) → O_2_ + NBT (purple)

$$4{\text{H}}^{+}\;+\;{\text{O}}_{2}^{.}\;\overset{\text{ESOD}}{\to }\;{\text{H}}_{2}{\text{O}}_{2}$$

In the absence of SOD, the radical reacts with the detector. In the event that SOD is present, it competes with the detector for superoxide radical.

### Stock solution preparation

All stock solutions were prepared in 50mM potassium phosphate pH7.8.

1. Nitroblue tetrazolium salt (NBT) 1 × 10^−3^ M
2. Flavine adenine dinucleotide (FAD) 5.56 × 10^−5^ M and EDTA 0.11 mM (Prepared together for test and reference standard), 5.56 × 10^−5^ moles of oxygen was bubbled into the the combined solution of FAD and EDTA. Oxygen was prepared by the decomposition of 0.038g of Potassium trioxo chlorate (V) in the presence of 0.01g of Manganese (IV) dioxide as a catalyst).
3. Flavine adenine dinucleotide (FAD) 5.56 × 10^−5^ M (without EDTA for blank)

### Experimental Procedure

Blank: 50 μl of 50 mM potassium phosphate pH7.8, was added to 900 μl of 5.56 × 10^−5^ M FAD (without EDTA) and 50 μl of 1 × 10^−3^ M NBT to give a final concentration of 5.0 × 10^−5^ M FAD and 5.0 × 10^−5^ M NBT.

### Reference Standard

50 μl of 50 mM potassium phosphate pH7.8, was added to 900 μl of 5.56 × 10^−5^ M FAD (with EDTA containing bubbled oxygen) and 50 μl of 1 × 10^−3^ M NBT to give a final concentration of 5.0 × 10^−5^ M FAD and 5.0 × 10^−5^ M NBT.

The blank was used to set double beam spectrophotomer to zero and change in absorbance at 550 nm was recorded.

### Test

Graduated volume of the sample was added to give 50 μl with 50 mM potassium phosphate pH7.8 Then 900 μl of 5.56 × 10^−5^ M FAD (with EDTA containing bubbled oxygen) and 50 μl of 1 × 10^−3^ M NBT were to give a final concentration of 5.0 × 10^−5^ M FAD and 5.0 × 10^−5^ M NBT. The blank was used to set double beam spectrophotometer to zero and change in absorbance at 550 nm was recorded. Amount of enzyme protein to inhibite the reaction between NBT and superoxide radical by 50% was determined. Activity per mg protein of ESOD was then calculated.

DEFINITION OF A UNIT: A unit is defined as the quantity of SOD required to inhibit reaction between O_2_^−^ and NBT by 50%.

### Protein estimation

Estimation of protein concentration was carried out by the method of Bradford (16). Bradford reagent for protein determination was prepared by dissolving 100mg of Coomassie Brilliant Blue in 50 ml of 95% ethanol, 100ml of 85% phosphoric acid and made up to a litre with distilled water. The reaction was initiated by adding 8 μl of sample to 320 μl of Bradford reagent and 30 μl of distilled water, protein concentrations were determined from a calibration curve using Bovine Serum Albumin (BSA) as standard by measuring the absorbance at 595 nm after 10mins incubation at 30°C.

### Statistical Analysis

Data are expressed as mean ± SD. In preliminary analyses, we used one-way analysis of variance (ANOVA) (SSPS 14.2 software) for continuous variables, to compare data from the three groups analyzed. Statistical significance levels of at least *p*<0.05 were used for all tests.

## RESULTS

### CD_4_ counts

Tables 1 and 2 show the multivariate analysis using ANOVA of biochemical indices in comparison to CD_4_ status of the subjects studied. The F values for MDA, vitamin A and ESOD were 43.16, 40.88 and 101.60 respectively (*p*<0.000).

### Lipid Peroxidation Products (MDA)

**Figure 1:**
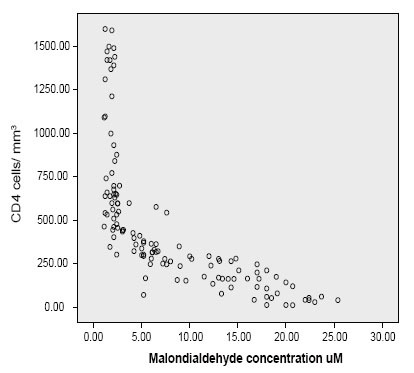
Correlation between serum malondialdehyde concentration and CD_4_ count.

The Plasma levels of MDA expressed as μmol/L are shown in Table 1. There was a significant reduction in Plasma MDA in patients with CD_4_ between 200−400 cells/mm^3^ on treatment compared with CD_4_ below 200 cells/mm^3^ naïve patients (Mean ± SD 8.93 ± 4.45, 95% CI 6.90−10.96 μM Vs 15.93 ± 6.93, 95% CI 13.01−18.85 μM; *p*<0.005) respectively. However there was no statistical difference between MDA plasma level between patients with CD_4_ count below 200 for treated and untreated groups (Mean ± SD17.68 ± 3.04 μM; 95% CI 15.92-19.43 μM Vs 15.93 ± 6.93 μM, 95% CI 13.01−18.85 μM) respectively. Figure 1 shows the correlation between CD_4_ count and plasma MDA concentration. There was a negative correlation between CD_4_ count status and serum MDA concentration [Pearson r=−0.68, *p*<0.01]. Plasma MDA concentration increased with the decrease in CD_4_ count.

**Table 1:** CD_4_ count status and serum levels of extracellular superoxide dismutase enzyme, Vitamin A and Malondialdehyde in HIV patients


Control				
CD_4_	Number	MDA (μM)	ESOD (U/mg serum protein)	Vitamin A (IU)
1146 ± 338 (600−)	20	1.84 ± 0.40^a^^c^	494.30 ± 117.84^a^^c^	47.40 ± 6.41^a^^c^
Treated				
CD_4_	Number	MDA (μM)	ESOD (U/mg serum protein)	Vitamin A (IU)
126 ± 52 (0−200)	14	17.68 ± 3.04	48.64 ± 23.80	17.86 ± 2.14
301 ± 51 (201−400)	21	8.93 ± 4.45^a^^c^	100.95 ± 32.38^b^^d^	28.81 ± 6.87^a^^c^
506 ± 71 (401−600)	12	3.04 ± 1.41^a^^c^	321.41 ± 86.64^a^^c^	45.25 ± 12.98^a^^c^
675 ± 82 (601−800)	6	1.95 ± 0.42^a^	436 ± 114.44^a^^c^	45.83 ± 4.92^a^^c^
Untreated				
CD_4_ counts	Number	MDA (μM)	ESOD (U/mg serum protein)	Vitamin A (IU)
88 ± 60 (0−200)	19	15.93 ± 6.06	34.21 ± 23.06	18.68 ± 3.58
297 ± 45(201−400)	16	7.13 ± 3.44^a^^c^	110.31 ± 26.30^b^^d^	28.06 ± 8.61^a^^c^
495 ± 59(401−600)	10	2.82 ± 1.87^a^^c^	233.30 ± 54.78^a^^c^	41.30 ± 9.25^a^^c^
957 ± 401 (601−	5	1.64 ± 0.57^a^^c^	524.20 ± 111.71^a^^c^	61.60 ± 15.27^a^^c^

Results are Mean ± SD.

a *p*<0.001 compared with untreated with CD_4_ count 88 ± 60;

b *p*<0.05 compared with untreated with CD_4_ count 88 ± 60;

c *p*<0.001 compared with treated with CD_4_ count 126 ± 52;

d *p*<0.05 compared with treated with CD_4_ count 126 ± 52.

### Plasma Vitamin A

**Figure 2:**
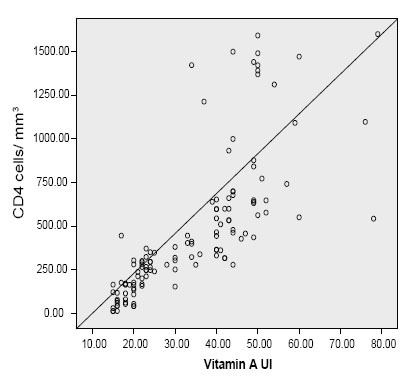
Correlation between Plasma Vitamin A concentration and CD_4_ Count.

The serum levels of vitamin A expressed as IU are shown in Table 1. There was a significant increase in the serum level of vitamin A with increase in CD_4_ count (Tables 1 and 2). The level of vitamin A in HIV treatment naïve patients with CD_4_ count 200-400 cells/mm^3^ was significantly higher than the same group of patients with CD_4_ count ≤200 cells/mm^3^ [28.06 ± 2.15 IU, 95% CI 23.47−32.65 IU Vs 18.68 ± 3.58 IU, 95% CI 16.96−20.40 IU, *p*<0.05] respectively. There was a positive correlation between serum Vitamin concentration and CD_4_ count [Pearson r=0.71 *p*<0.01] (Fig. 2). However there was a negative correlation between plasma malondialdehyde and vitamin A concentration (Pearson r=−0.75; *p*<0.01). There was a positive correlation between serum vitamin A concentration and ESOD activity (Pearson r=0.79; *p*<0.01).

**Table 2:** Multivariant analysis of biochemical indicators in comparison to CD_4_ status

ANOVA						
		Sum of Squares	df	Mean Square	F	Sig.

MDA (μmol/L)	Between Groups	4281.305	8	535.163	43.155	0.0000
	Within Groups	1413.700	114	12.401		
	Total	5695.004	122			
VitA (IU)	Between Groups	19516.285	8	2439.536	40.879	0.0000
	Within Groups	6803.178	114	59.677		
	Total	26319.463	122			
ESOD (U/mg serum protein)	Between Groups	3927377.855	8	490922.232	101.569	0.0000
	Within Groups	551004.779	114	4833.375		
	Total	4478382.634	122			

### Serum Extracellular superoxide dismutase

**Figure 3:**
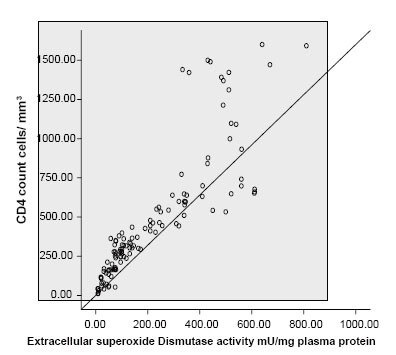
Correlation between extracellular superoxide dismutase activity and CD_4_ count.

The levels of extracellular superoxide dismutase activity are shown in Table 1. The activity of extracellular dismutase showed a similar pattern with that of vitamin A in relation to CD_4_ counts. There was a positive correlation between extracellular superoxide dismutase and CD_4_ count in this study (Pearson r=0.86, *p*<0.01] (Fig. 3). There was also a negative correlation between serum malondialdehyde concentration and extracellular superoxide dismutase activity (Pearson r=−0.71, *p*<0.01). The change in extracellular SOD activity was not statistically significant for naïve and treated HIV subject with CD_4_ ≤200 (34.21 ± 23.06 mU/mg serum protein; 95% CI 23.10−45.33 06 mU/mg serum protein Vs 48.64 ± 23.80 mU/mg serum protein; 95% CI 34.90-62.39; *p*<0.1). There was very slight significant increase in ESOD activity between HIV1 treatment-naïve and treated HIV 1 patients with CD_4_ count 200−400 (110.31 ± 26.30 mU/mg serum protein, 95% CI 96.30−124.32 mU/mg serum protein Vs 100.95 ± 32.38 mU/mg serum protein 95% CI 86.21−115.69 mU/mg serum protein *p*<0.05). There was a significant increase in ESOD activity with CD_4_ count 200-400 over patients with CD_4_ count ≤200 for either HIV treatment naïve or treated HIV subjects (*p*<0.001).

## DISCUSSION

The success of HIV infection management does not only rely on effective treatment, but also on efficient and cost effective monitoring tests. In Nigeria the cost of drugs per patients was about $368, while monitoring was about $170, representing 46% of drug cost in 2004. Though government subsidized ARV drugs, patients paid about $86 annually as a contribution to the drug cost (17). Furthermore, the monitoring and screening costs ($170) were borne exclusively by the patients, bringing total patient out-of-pocket expenditure up to $256 per year. This is equivalent to almost 75 percent of annual per capita gross domestic product, well beyond the resources of most Nigerians (17). The development of an effective ARV program therefore, must include support not only for ARV drugs but for all aspects of patient cost (17).

ARVs were being administered in only 25 treatment centers across the country which was a far from adequate attempt at helping the estimated 550,000 people requiring antiretroviral therapy (17). As a result, in 2006 Nigeria opened up 41 new AIDS treatment centers and started handing out free ARVs to those who needed them most. Treatment support between 2006-7 was impressive, rising from 81,000 people (15% of those in need) to 198,000 (26%) by the end of 2007. Despite this progress, Nigeria is still yet to attain the target of providing universal access for AIDS treatment. The HIV prevalence in Nigeria represents 3.6% of adult aged 15–49years (18). This translates into 2,600,000 people infected with HIV (19). There are currently 552,000 people in the country who do not have access to the ARV treatment that they need (10). There is a large reservoir of basic information that suggests a strong association between HIV infection and oxidative stress (1, 20, 21). Since the brunt of the monitoring testing for both patients who are eligible and non-eligible for treatment based on the CD_4_ status criterion is taken by the patients, it is thought possible to evaluate other surrogate biochemical tests in evaluating CD_4_ status in patients who are not eligible for therapy in resource constrained facilities in the rural and urban areas. Similarly, subjects who are eligible for treatment but do not have the wherewithal could also benefit from using the biochemical parameters which are surrogate markers of CD_4_ count. The use of MDA and extracellular ESOD assay correlates negatively and positively significantly with CD_4_ count respectively (Fig. 1 and Fig. 3). Increase in ESOD was accompanied by a corresponding increase in CD_4_ status (Fig. 3). Conversely increase in MDA concentration was associated with decrease in CD_4_ count (Fig. 1). Though vitamin A status can be influenced by intake, a positive correlation has been observed between Vitamin A concentration and CD_4_ count (Fig. 2), with a negative correlation with MDA concentration. In this study, deliberate attempt was made to use simple and cheap methods, in order to suit our resource constrained health facilities as well as our current energy predicaments where constant power supply has become a nightmare to our nation. As the reagents can be prepared just before use, this may obviate the need of storing highly expensive reagents in refrigerators with extremely low temperature. Though highly active antiretroviral therapy has been reported to be associated with increased oxidative stress (22), in this study the increase in oxidative stress for patients with CD_4_ count between 200-400 cells/mm^3^ between treated and untreated was not significant (MDA 8.93 ± 4.45 μmol/L Vs 7.13 ± 3.44 μmol/L *p*<0.13) respectively (Table 1). Similarly, the increased level of MDA in treated for patients with CD_4_ count 401-600 cells/mm^3^ was not significant compared with untreated with the same level of CD_4_ count (3.04 ± 1.41mmol/L Vs 2.82 ± 1.87 mmol/L, *p*=0.088) (Table 2).

## CONCLUSION

We have shown that oxidative stress increases with depression of CD_4_ count in HIV infection. Thus the use of oxidative stress and antioxidants indicators can be used as surrogate markers of CD_4_ count in the laboratory for monitoring of HIV treatment-naïve patients in laboratory with facilities constrains.
